# Differential analysis of the bacterial community in colostrum samples from women with gestational diabetes mellitus and obesity

**DOI:** 10.1038/s41598-021-03779-7

**Published:** 2021-12-21

**Authors:** J. S. Gámez-Valdez, J. F. García-Mazcorro, A. H. Montoya-Rincón, D. L. Rodríguez-Reyes, G. Jiménez-Blanco, M. T. Alanís Rodríguez, R. Pérez-Cabeza de Vaca, M. R. Alcorta-García, M. Brunck, V. J. Lara-Díaz, C. Licona-Cassani

**Affiliations:** 1 Tecnológico de Monterrey, Escuela de Ingeniería y Ciencias, Ave. Eugenio Garza Sada 2501 sur, Monterrey, NL 64849 México; 2 Research and Development, MNA de México, San Nicolás de los Garza, NL México; 3 Tecnológico de Monterrey, Escuela de Medicina y Ciencias de la Salud, Monterrey, NL México; 4 Coordinación de Investigación y División de Investigación Biomédica, C.M.N. “20 de Noviembre”, ISSSTE, Ciudad de México, México; 5 Departamento de Neonatología, Hospital Regional Materno Infantil, Servicios de Salud de Nuevo León, Guadalupe, México; 6 Division of Experimental Medicine, The Institute for Obesity Research, Tecnológico de Monterrey, Monterrey, NL México; 7 Division of Integrative Biology, The Institute for Obesity Research, Tecnológico de Monterrey, Monterrey, NL México

**Keywords:** Clinical microbiology, Microbiology, Microbial communities, Clinical microbiology

## Abstract

Gestational Diabetes Mellitus (GDM) and obesity affect the functioning of multiple maternal systems and influence colonization of the newborn gastrointestinal through the breastmilk microbiota (BMM). It is currently unclear how GDM and obesity affect the human BMM composition. Here, we applied 16S-rRNA high-throughput sequencing to human colostrum milk to characterize BMM taxonomic changes in a cohort of 43 individuals classified in six subgroups according to mothers patho-physiological conditions (healthy control (n = 18), GDM (n = 13), or obesity (n = 12)) and newborn gender. Using various diversity indicators, including Shannon/Faith phylogenetic index and UniFrac/robust Aitchison distances, we evidenced that BMM composition was influenced by the infant gender in the obesity subgroup. In addition, the GDM group presented higher microbial diversity compared to the control group. *Staphylococcus*, *Corynebacterium* 1, *Anaerococcus* and *Prevotella* were overrepresented in colostrum from women with either obesity or GDM, compared to control samples. Finally, *Rhodobacteraceae* was distinct for GDM and 5 families (*Bdellovibrionaceae*, *Halomonadaceae*, *Shewanellaceae*, *Saccharimonadales* and *Vibrionaceae*) were distinct for obesity subgroups with an absolute effect size greater than 1 and a q-value ≤ 0.05. This study represents the first effort to describe the impact of maternal GDM and obesity on BMM.

## Introduction

Breastfeeding during the first semester of life is crucial, since it provides all the nutritional, cellular and microbial requirements for the newborn’s metabolic, immunological, and neurological development^1–4^. Human breastmilk contains a microbial population (microbiota) of approximately 10^6^ bacterial cells/mL and about 2000 Amplicon Sequence Variants (ASVs)^5,6^. Maternal conditions (ethnicity, diet, body mass index (BMI), health), delivery type and neonate gender have been correlated with variations in the relative abundance of taxa and diversity of the breastmilk microbiota (BMM)^5,7–9^. Since it has been shown that a compromised BMM signature affects the newborn´s future health^1,3^, it is necessary to study BMM changes in the context of maternal pathophysiology.

Obesity (BMI ≥ 30 kg/m^2^) is one of the most common diseases affecting pregnancies and lactation worldwide, with almost one third of women of reproductive age suffering from this condition^10^. Adverse effects in mothers include metabolic alterations, pre-eclampsia, gestational hypertension, and depression^11^. Having an increased BMI during pregnancy increases 2–8 times the risk of developing Gestational Diabetes Mellitus (GDM)^11–13^. GDM is characterized by a chronic hyperglycemia recognized during the gestational period and has a global incidence of 15%^14^. Women with GDM present decreased insulin sensitivity and β-cell dysfunction, which increases the risk of developing type 2 diabetes, and metabolic syndrome and cardiovascular disease after pregnancy^15,16^.

Several studies have focused on differentiating the breastmilk bacterial signature of obesity. For instance, preconception obesity has been related to a higher prevalence of Firmicutes and *Staphylococcus* and a decrease in Proteobacteria, *Bifidobacterium* and *Streptococcus* abundances in BMM that can cause aberrant neonate gut colonization and an increased risk of altered metabolism maturation^17–20^. While no research of BMM of patients with GDM has been reported, variations in fecal, oral, amniotic fluid and vaginal microbiota show an overrepresentation of members of the *Prevotellaceae* family^21,22^. Obesity and GDM are considered the bottom-line of a trans-generational vicious cycle of bacterial disequilibrium affecting the entire population. While strongly correlated, it is of great importance to differentiate the BMM composition under the pathological conditions of obesity and GDM. Most importantly, independent changes are key for the prospective design of personalized probiotics and therapies.

Here, we report a differential analysis of the composition of the microbiota in colostrum samples of Mexican mothers affected with obesity or GDM. Our study cohort includes mothers with obesity (≥ 30 kg/m^2^, non-GDM) and mothers with GDM (< 30 kg/m^2^, non-obese) from Monterrey, the metropolitan area with the highest prevalence of GDM of all major cities in Mexico. We sampled colostrum within the first 24 h after birth and used 16S rRNA amplicon sequencing to quantitatively characterize the microbial diversity of samples under each physiopathology compared to healthy donors. This work is the first descriptive analysis to differentiate the BMM present in the colostrum of mothers with GDM or obesity.

## Results

Colostrum samples from 43 Mexican mothers, aged 20–32 years were used in this study. All in-hospital deliveries were performed in accordance with Mexican guidelines and the gestation periods lasted an average of 39.4 weeks. A total of 18 samples from mothers with BMI < 25 kg/m^2^ (non-obese) and no GDM indicators were considered as controls. Twelve milk samples were collected from obese mothers (BMI ≥ 30 kg/m^2^) without GDM, and thirteen samples were taken from mothers with GDM without obesity (eight overweight—but not obese-and five lean). Among GDM participants, all were diet controlled, none of them were treated with Metformin but four patients received insulin treatment. Most of the participants were multiparous and 24 deliveries occurred by cesarean section. As standard surgical procedure, peripartum antibiotics were given in 25 deliveries. Neonate sex distribution was 48.8% male and 51.2% female. All clinical and demographic data are summarized in Table 1.

**Table 1:** Clinical characteristics of subjects included in the study (n = 43).

	n	Value
**Maternal age (year)**	43	24.6 ± 3.4
**Maternal BMI (kg/m**^**2**^**)**		
Non-obese (20–29.9)	31	23.8 ± 2.6
Obese (≥ 30)	12	33.2 ± 3.0
**Gestational age (weeks)**		
Term	43	39.4 ± 1.3
Preterm	0	0.0 ± 0.0
**Problems during pregnancy**		
None	22	51.2%
Gestational diabetes (insulin treated)	13 (4)	30.2% (9.3%)
Urinary tract infection	9	20.9%
Cervicovaginitis	6	14.0%
**Mode of delivery**		
Vaginal	19	44.2%
Cesarean	24	55.8%
**Parity**		
1	10	23.3%
≥ 2	33	76.7%
**Exposure to antibiotics**		
3 months prior delivery (during delivery)	0 (25)	0% (58.1%)
None	18	41.9%
**Sex of the baby**		
Female	22	51.2%
Male	21	48.8%

**Figure 1:**
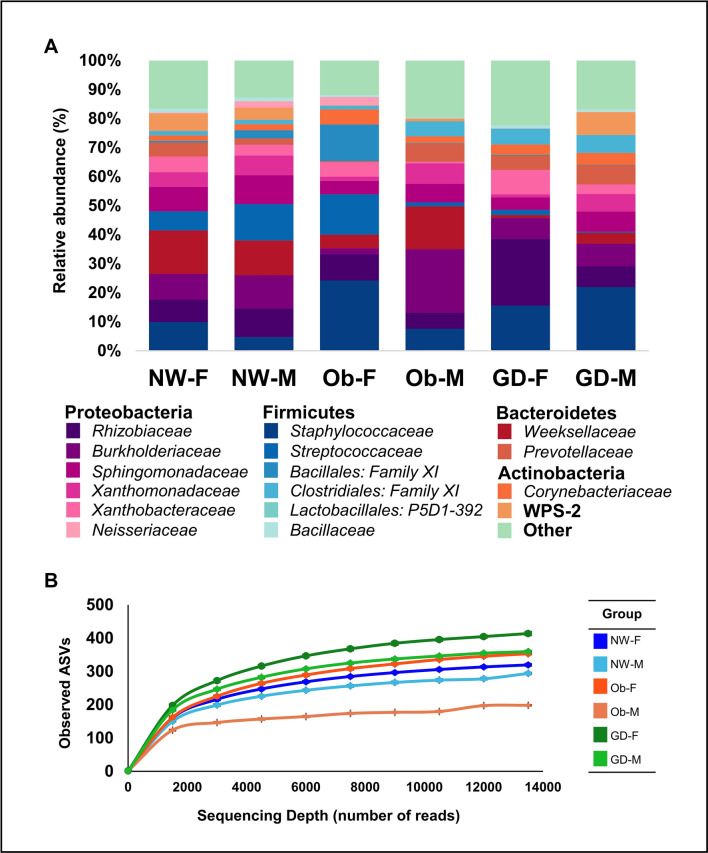
Bacterial diversity of colostrum samples. (**A**) Taxonomic profile at family level divided by study subgroups (maternal health condition and sex of the newborn). (**B**) Rarefaction curves from subgroups of colostrum samples relating the sequencing depth and the estimated number of bacteria. *ASVs* amplicon sequence variants, *NW-F* healthy normal weight-female (n = 8), *NW-M* healthy normal weight-male (n = 10), *Ob-F* obesity positive GDM negative-female (n = 8), *Ob-M* obesity positive GDM negative-male (n = 4), *GD-F* GDM positive obesity negative-female baby (n = 6), *GD-M* GDM positive obesity negative-male baby (n = 7).

We collected the colostrum samples within the first 24 h after birth and stored them at -20 °C until analysis. The 16S amplicon sequencing yielded 1,675,157 high quality reads with a mean number of 38,957 ± 27,723 reads per sample. Using the latest version of Silva 132 database (confidence identity score of 99%), our dataset includes a total of 1,496 amplicon sequence variants (ASVs) assigned to 30 phyla, 58 classes, 133 orders, 217 families, 395 genera and 335 species. In addition to eliminating rare taxa as possible contaminants (≤ 25 reads in total), we performed a batch analysis for all DNA extractions^23^. As part of this analysis, we identified batch effects for *Pseudomonas, Enterobacteriaceae, Ralstonia* and *Herbaspirillum* across our dataset. Even though *Pseudomonas* and *Enterobacteriaceae* form part of other breastmilk microbial compositional studies^5,8,24,25^, we decided to remove these from our dataset in order to lower the bias of further analysis and correlations. We classified the dataset into six study subgroups according to the pathophysiological condition (diabetes or obesity) of the mother and the newborn’s gender (male or female): obesity-female (Ob-F; n = 8); obesity-male (Ob-M; n = 4); GDM-female (GD-F; n = 6); GDM-male (GD-M; n = 7), healthy normal weight-female (NW-F; n = 8) and healthy normal weight-male (NW-M; n = 10). In order to simplify the analysis, we identified taxonomic differences at the levels of family (Fig. 1) and genera (Fig. 3 and Supplementary Table S2), according to all of the study groups.

Overall, the samples were overrepresented by *Staphylococcaceae*, with a relative abundance mean of 13.9% ± 16.5% (range 0.1–76.3%), followed by the members of the Proteobacteria phylum *Rhizobiaceae* (relative abundance mean of 10.3% ± 11.1%; range 0.1–47.2%) and *Burkholderiaceae* (9% ± 11.7%; range 0–48.6%). Less-represented taxa were the Bacteroidetes members *Weeksellaceae* (8.6% ± 11.9%; range 0–37.2%), *Prevotellaceae* (3.8% ± 7.1%; range 0–24.3%); and *Corynebacteriaceae* (3.2% ± 4.5%; range 0–16.2%) (Fig. 1A). The group “Other” represents phyla with less than 1% of the total relative abundance (Fig. 1A). Detailed information about the abundance of ASVs found per sample is presented as Supplementary Table S1.

### Diversity indicators suggest distinct microbial community signatures for gestational diabetes, obesity and newborn gender subgroups

**Figure 2:**
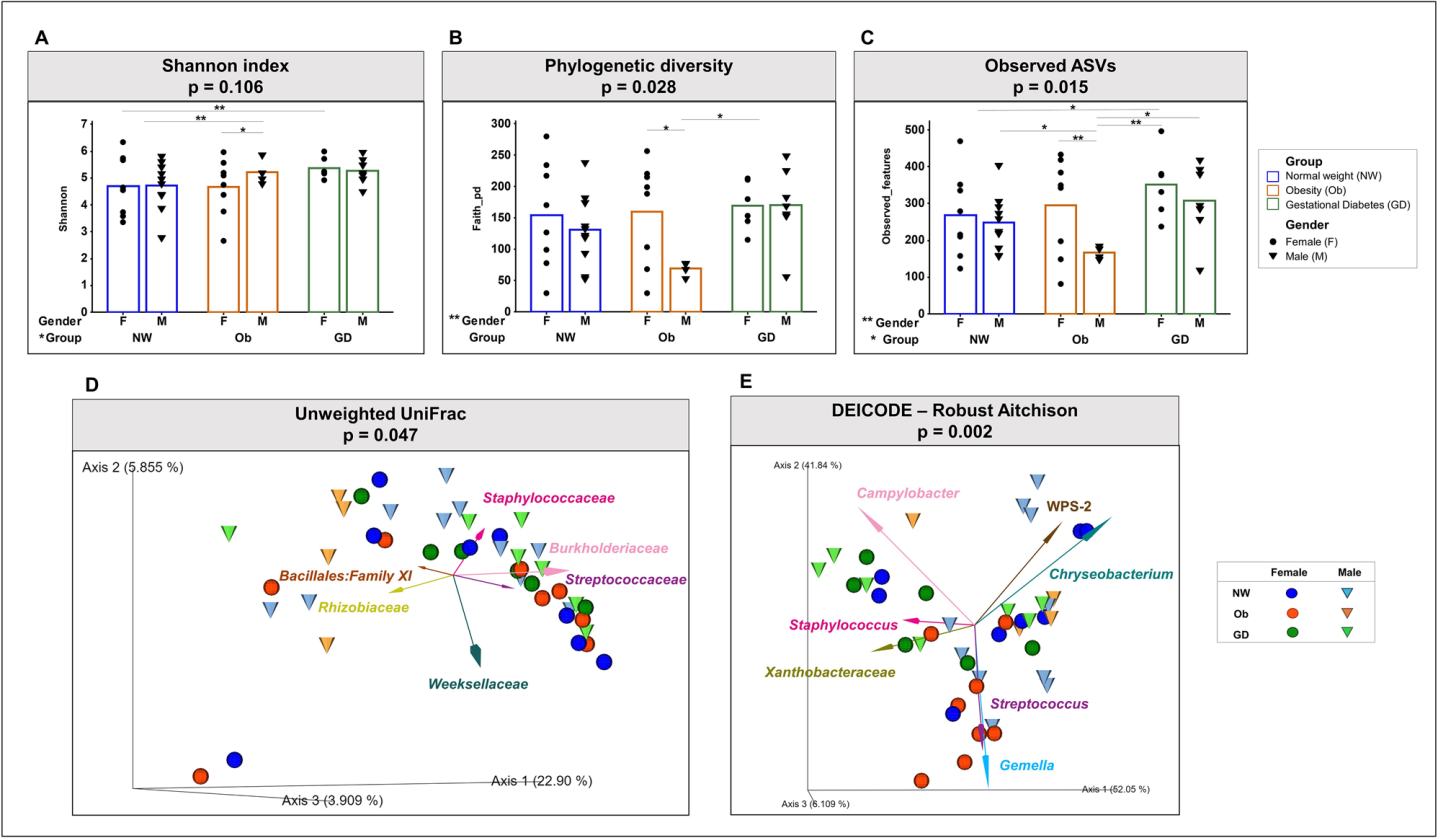
Alpha and beta diversity indexes of colostrum (**A**–**C**) Alpha diversity. (**D**, **E**) Beta diversity. (**A**) Shannon index. (**B**) Number of observed ASVs. (**C**) Phylogenetic diversity. All of the Alpha indexes showed significant differences after a general linear model (glm) with a confidence level of 95% (p ≤ 0.05). The Fisher test was implemented for comparisons. Graphs were plotted using GraphPad Prism version 6.0.0, GraphPad Software, San Diego, California USA (www.graphpad.com). (**D**) Unweighted principal coordinate analysis (PCoA) biplot of UniFrac distances with vectors at family level. (**E**) Robust principal component analysis (RPCA) biplot using DEICODE (robust Aitchison). Beta diversity distance matrices were generated and analyzed in the QIIME2 platform. Beta indexes showed significant differences after being assessed by permutational ANOVA (999 permutations). *p ≤ 0.10; **p ≤ 0.05. Plots were visualized using the QIIME2 plugin Emperor^26^.

We used a general linear model (GLM) using alpha diversity metrics at a sequencing depth of 6130 in order to quantify the influence of GDM, obesity, antibiotic exposure, multiparity and newborn gender (data not shown). Among the parameters tested, health condition (GDM and obesity) and peripartum antibiotic exposure showed a statistically significant association (p ≤ 0.10) with the Shannon index and observed ASVs. Use of peripartum antibiotics was related to a decrease in microbial diversity. On the other hand, GDM presented the highest values in alpha indexes. Newborn gender showed a statistically significant association (p ≤ 0.05) with phylogenetic diversity and observed ASVs (Fig. 2A–C), with the all-female subgroups more diverse than their male counterparts. Fisher analysis showed statistically significant differences between NW-F and GD-F subgroups. Finally, the colostrum samples from the Ob-M presented the lowest levels of alpha diversity and differed statistically from all the other subgroups, including its direct female counterpart (Fig. 2A–C). Our statistical analysis disregarded delivery mode as a possible background contamination as we did not see statistical significance for alpha or beta diversity (data not shown).

We also estimated microbiome beta diversity using unweighted UniFrac distance (Fig. 2D). Our results show that the Ob-M subgroup clusters separately from the rest of the samples (PERMANOVA; p = 0.047; 999 permutations). Using the unweighted distance matrix, we generated a PCoA biplot in order to show that clustering was significant for the Ob-M (p < 0.05), compared to NW-M subgroup. Arrows in the plot represent the correlation at family level with the PCoA axes, indicating their contribution to the variation (Fig. 2D). While samples from the GD-F, GD-M, NW-F and Ob-F subgroups show high similarity in terms of microbial composition, the unweighted measurement indicates that there is a phylogenetic difference between the Ob-M and the rest of the subgroups (p < 0.05).

In order to study the compositional nature of the dataset, we used the beta-diversity compositional Aitchison distance (PERMANOVA; p = 0.002; 999 permutations) (Fig. 2E). The robust principal component analysis (RPCA) shows that the samples do not cluster according to their corresponding subgroup association. Only the PERMANOVA tests and pairwise comparisons showed that Ob-M was different to both GDM subgroups (p < 0.05) and that the Ob-F differed significantly from its male counterpart (Ob-M) and both the healthy and GDM subgroups (p < 0.05). In addition, the GD-M subgroup differed, although not significantly so, from its healthy counterpart (NW-M; p < 0.10). The seven taxa presented as vectors in the plot are the most significant drivers of sample location (Fig. 2E).

### The microbial community compositional core and distinctive signatures among healthy-normal weight, obesity and gestational diabetes subgroups

We defined the colostrum core microbiota as taxonomic families present in all samples with a minimum 1% of total mean relative abundance. Eight families were identified as core taxa and comprised 56.8% ± 11.3% of the total (Table 2). The most abundant taxa were *Staphylococcaceae* with a general mean of 13.9% ± 16.5%, followed by *Rhizobiaceae* (10.3% ± 11.1%), *Burkholderiaceae* (9% ± 11.7%) and *Streptococcaceae* (7.1% ± 16.1%). These results demonstrate the high variability of the core bacteria among subgroups and individuals. The four most abundant families belonging to the core were found to describe most of the variation in the ordination space observed in the unweighted PCoA biplot, and were represented as arrows (Fig. 2D). However, no clear participation of families to the formation of subgroups was visualized with the implementation of UniFrac metrics.

**Table 2:** Colostrum core microbiota at the taxonomic family level (% relative abundance ± standard deviation).

Core family	Overall (%)	Healthy-female (NW-F)	Healthy-male (NW-M)	Obesity-female (Ob-F)	Obesity-male (Ob-M)	GDM-female (GD-F)	GDM-male (GD-M)
*Staphylococcaceae*	13.9 ± 16.5	9.9 ± 14.9	4.7 ± 4.6	24.2 ± 25.8	7.6 ± 7.0	15.6 ± 11.9	21.9 ± 16.9
*Rhizobiaceae*	10.3 ± 11.1	7.7 ± 3.7	9.8 ± 12.6	9.1 ± 11.7	5.5 ± 2.8	22.9 ± 16.6	7.1 ± 4.5
*Burkholderiaceae*	9.0 ± 11.7	8.8 ± 9.0	11.5 ± 14.7	2.0 ± 4.5	21.9 ± 9.5	7.2 ± 15.4	7.8 ± 9.3
*Streptococcaceae*	7.1 ± 16.1	6.6 ± 17.1	12.5 ± 23.4	13.9 ± 19.3	1.3 ± 1.9	1.8 ± 1.5	0.4 ± 0.2
*Sphingomonadaceae*	7.1 ± 6.3	8.4 ± 6.5	10.0 ± 7.2	4.6 ± 6.2	6.4 ± 3.9	4.3 ± 2.2	6.9 ± 8.0
*Xanthomonadaceae*	4.6 ± 6.1	5.1 ± 4.4	6.8 ± 8.1	1.5 ± 2.4	7.1 ± 5.5	1.1 ± 1.1	6.1 ± 8.6
*Prevotellaceae*	3.8 ± 7.1	4.8 ± 8.2	2.2 ± 4.5	0.4 ± 0.4	6.1 ± 10.9	4.9 ± 8.0	6.8 ± 10.0
*Bacillaceae*	1.0 ± 1.5	1.4 ± 3.0	1.3 ± 1.7	0.8 ± 0.7	0.4 ± 0.2	1.0 ± 0.6	0.9 ± 0.5
Total relative abundance (%)	56.8 ± 11.3	52.7 ± 9.5	58.8 ± 11.8	56.4 ± 14.2	56.3 ± 8.3	58.8 ± 11.5	58.0 ± 10.4

### Microbial composition of colostrum samples is influenced by newborn gender

**Figure 3:**
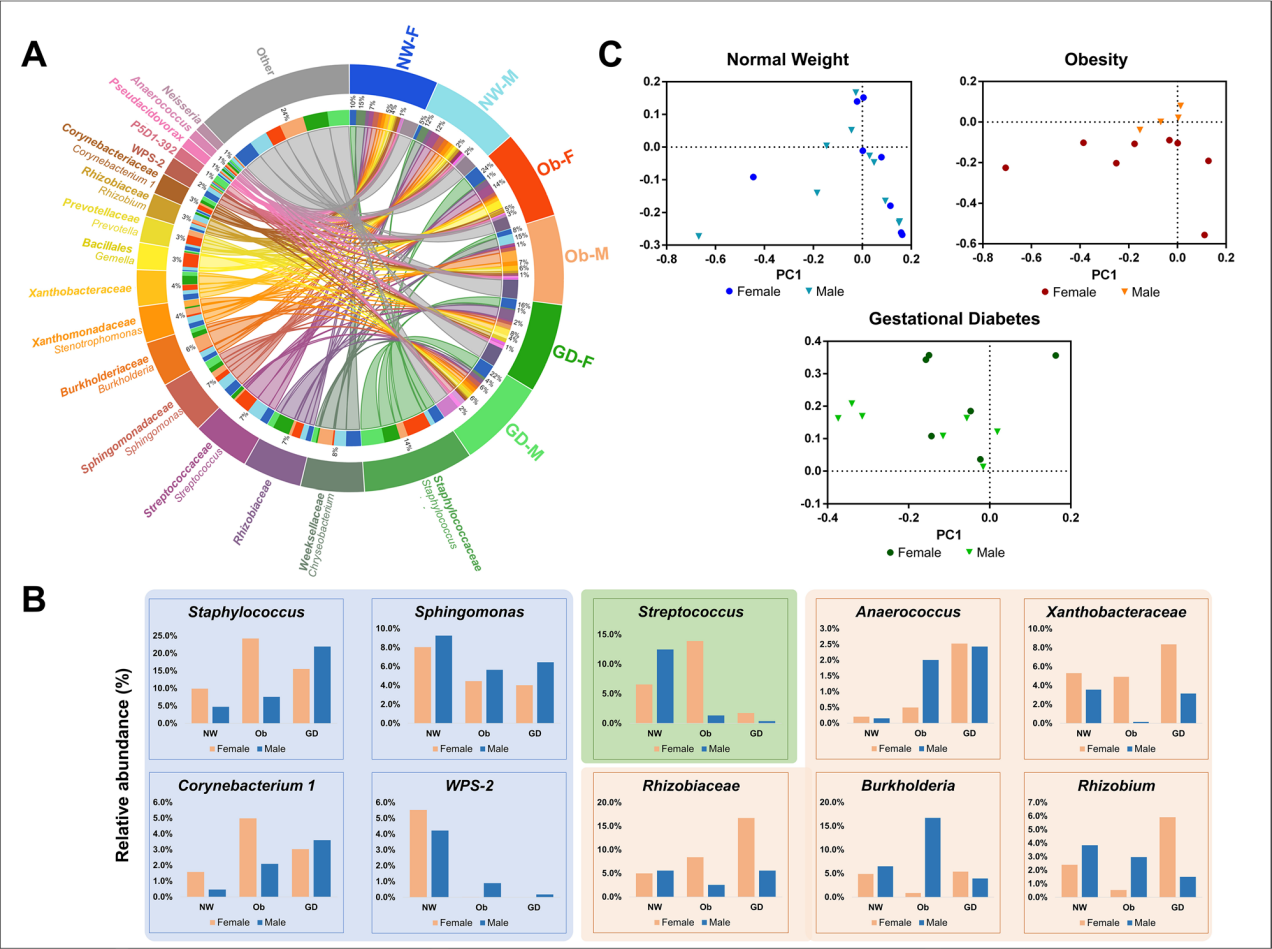
Colostrum taxonomic profile at genera level. (**A**) CIRCOS representation of the most abundant genera amongst healthy, obesity and GDM subgroups. “Other” represents all the taxa with less than 1% of total abundance. Circos plot was created with Circa (http://omgenomics.com/circa). (**B**) Relative abundance of the genera that differ by group. (**C**) Principal Component Analysis (PCA) of the difference in taxa by newborn’s sex in each experimental group. In all cases the first two components (contribution ≥ 80% of variance) were plotted using GraphPad Prism version 6.0.0, GraphPad Software, San Diego, California USA (www.graphpad.com). *NW-F* healthy normal weight-female, *NW-M* healthy normal weight-male, *Ob-F* obesity positive GDM negative-female, *Ob-M* obesity positive GDM negative-male, *GD-F* GDM positive obesity negative-female baby, *GD-M* GDM positive obesity negative-male baby.

We observed gender bias in the taxonomic composition of the samples of our study (Fig. 3A and Supplementary Table S2). From the 18 most abundant taxonomic families identified, the healthy normal weight subgroup showed gender bias for six families, the GDM subgroup showed bias for eight families and the obesity subgroup showed gender bias for 15 families. The families *Chryseobacterium* and *Prevotella* were more abundant in the NW-F subgroup compared to its male counterpart (NW-M), while Ob-F and GD-F subgroups were less abundant. *Streptococcus* showed the opposite behavior i.e., a lower abundance for the NW-F compared to the male counterpart, while higher abundance was observed in the Ob-F and GD-F subgroups (p ≤ 0.10) compared to their male counterpart. The *Xanthobacteraceae* family was more abundant in the female subgroups for the three conditions (healthy normal weight, obesity and GDM; p < 0.10). The opposite pattern was observed for *Sphingomonas* and *Stenotrophomona*s in which male subgroups were more abundant than female subgroups. Figure 3C shows the PCA for the three subgroups in our study. p-values were shown when statistical significance was observed.

### Changes in relative abundance of colostrum microbiota of mothers with obesity and GDM

The control subgroup (NW) was more abundant for *WPS-2* (5.5%, female and 4.2%, male) and *Sphingomonas* (8%, female and 9.3%, male) compared to the obesity and GDM subgroups (p ≤ 0.10). On the other hand, *Corynebacterium 1*, *Anaerococcus* and *Staphylococcus* were more abundant in both obesity (p < 0.05 for the three genera) and GDM (p < 0.05 for *Staphylococcus* and p < 0.10 for *Corynebacterium 1*) subgroups, compared to the control group. *Prevotella* was significantly more abundant in the GDM subgroup compared to the NW (p < 0.10) and obesity (p < 0.05) subgroups. Unfortunately, the strong gender bias observed in the obesity subgroup masked significant changes in abundance for most of the members of the microbiota we studied. The only subgroup we observed with differences was Ob-F in which *Gemella* (p < 0.10) and *Staphylococcus* (p < 0.10) were higher than their healthy counterparts. While no statistical significance was observed, *Streptococcus* and *Neisseria* presented higher relative abundance in Ob-F compared to the rest of the subgroups (Fig. 3A and Supplementary Table S2).

We observed that *Streptococcus* was lower in GDM compared to the obesity (p < 0.05) and healthy normal weight subgroups (Fig. 3B). *Xanthobacteraceae* and *Rhizobiaceae* were more abundant in the GDM subgroup compared to that of obesity (p < 0.10). *Rhizobium* had the highest relative abundance in GD-F, compared to the Ob-F and NW-F subgroups (p < 0.05). *Burkholderia* was significantly higher in the Ob-M subgroup compared to the GDM (both female and male) subgroup (p < 0.05). Colostrum from subjects with GDM showed a higher abundance of “Other genera” (29.8% and 25.9% for female and male, respectively), suggesting that most of this contribution to the relative abundance of both subgroups is due to genera with less than 1% in total abundance (Fig. 3A and Supplementary Table S2). This is supported by the rarefaction curves, which reveal that the GD-F, GD-M and Ob-F had higher values of estimated number of observed ASVs (Fig. 1B). While four out of 13 participants with GDM received insulin treatment, we did not find any statistical significance at the level of alpha or beta diversity metrics.

**Figure 4:**
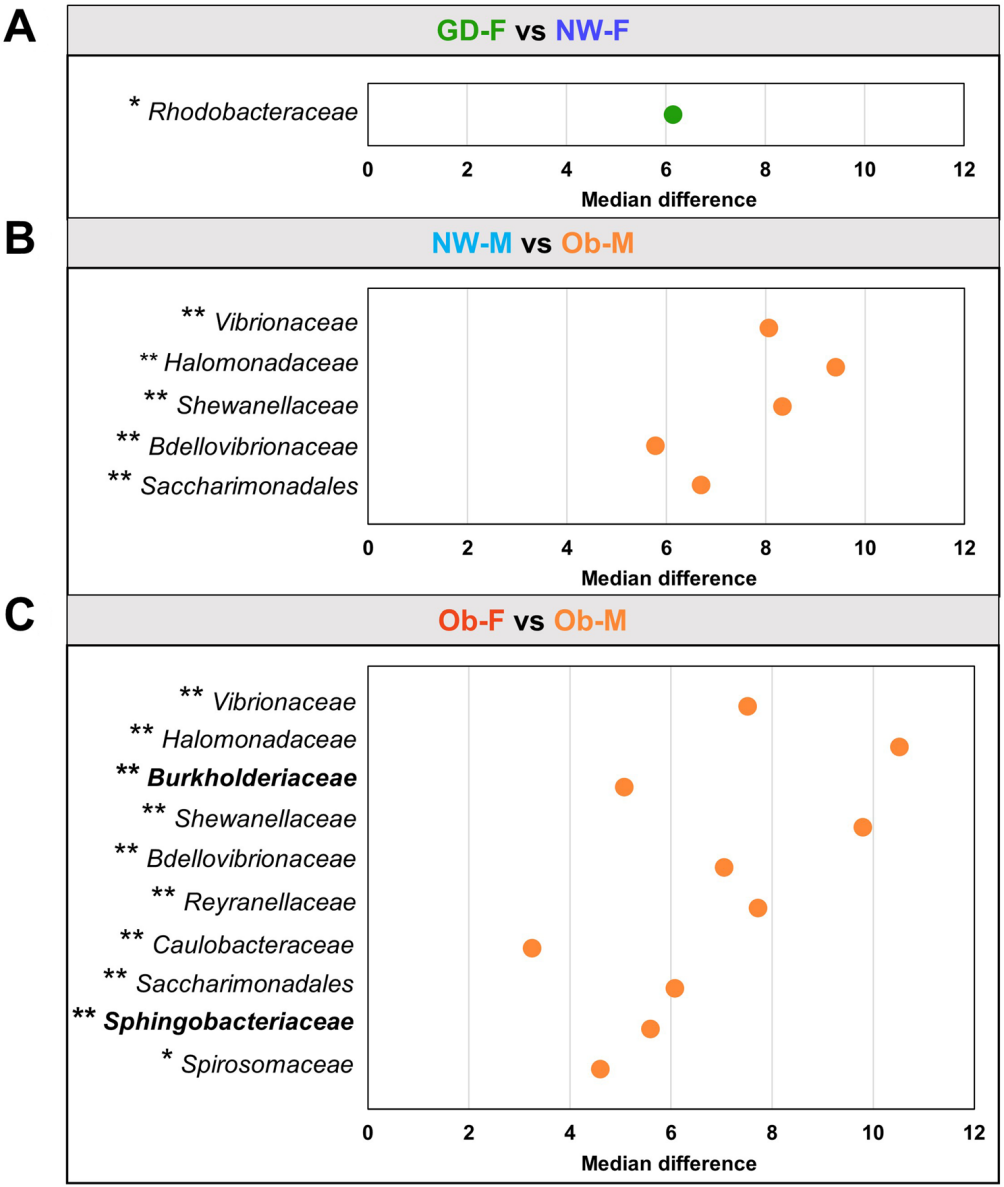
Differential bacteria at family level between subgroups. Each panel shows a plot illustrating differentially abundant taxon for each comparison and their median difference of centered log-ratio (clr) transformation, which indicates the dimension of the difference in abundance. Taxa with bold letters represent members of the core microbiota. Dots in the plot represent the median difference of significant features after a Welch’s t test (adjusted p-value ≤ 0.10) and effect size ≥ 1. Dots are colored according to the subgroup that contains the greater fraction. (**A**) Comparison of GDM-female versus healthy-female. (**B**) Comparison of healthy-male versus obesity-male. (**C**) Comparison of obesity-female versus obese-male. *q ≤ 0.10; **q ≤ 0.05.

Finally, we used the Aldex2 tool^27^ in order to obtain taxonomic differences (at the family level) between subgroups. We identified the taxa that were driving the difference among the subgroups and obtained effect plots (based on the effect size) that allowed us to visualize whether the variation was higher among or within subgroups. Given the high variability among samples, we only observed differentially abundant ASVs with a significant expected Benjamini–Hochberg corrected p-value of Welch’s t test (q ≤ 0.10) in three sample pairs (Fig. 4). In the GD-F vs NW-F comparison, the family *Rhodobacteraceae* was identified as different (q < 0.10; Fig. 4A). In the NW-M vs Ob-M we found *Vibrionaceae*, *Halomonadaceae*, *Shewanallaceae*, *Bdellovibrionaceae*, and *Saccharimonadales* with an absolute effect size greater than 1 and a q-value ≤ 0.05 (Fig. 4B). In the Ob-M vs Ob-F comparison, 10 significant taxa were different, of which only 2 (*Burkholderiaceae* and *Sphingobacteriaceae*) corresponded to the core families (Fig. 4C). Based on the median difference between subgroups, we observed that in all comparisons, the Ob-M subgroup had a significantly higher abundance for differential taxa.

## Discussion

We present the first compositional study of the colostrum microbiota of mothers affected by obesity or gestational diabetes mellitus. Colostrum is ideally the first postnatal maternal fluid in contact with the gastrointestinal system of the newborn; therefore, identifying differences in its microbial composition could be correlated with an aberrant infant gut colonization and further explain dysfunctions in the immune gastrointestinal system. Overall, we show that the microbial composition in our dataset is overrepresented with *Staphylococcaceae* and *Rhizobiaceae* families (Fig. 1A). *Staphylococcus*, *Chryseobacterium*, *Streptococcus* and *Sphingomonas* were the most abundant genera, which correlates with previous reports of women from Mexico, Guatemala, Taiwan, Finland and China (Beijing area)^8,24,28,29^. However, other datasets showed *Pseudomonas* and *Staphylococcus* as predominant taxa in breastmilk samples from Spanish and Irish individuals, in addition to commensal and obligate anaerobes such as *Bifidobacterium* and *Bacteroides*^25,30^.

In general, low-biomass samples are unstable and their relative abundance might be influenced by subtle differences in sample handling or sequencing methodologies^31,32^. More importantly, low-biomass samples are prone to DNA extraction and library preparation kit contaminants^23^. Different studies show that the presence of *Pseudomonas* and *Ralstonia* in breastmilk could be the result of reagent contamination effect, especially when culturomics approaches fail to isolate such microorganisms in selective culture media^30^. As an alternative to the utilization of negative controls, batch processing for all of the samples allows for discrimination of contaminants^23^. In our study, the samples were processed for DNA purification in a total of 16 batches. Detailed analysis allowed us to identify the batch effect across our dataset for *Pseudomonas, Enterobacteriaceae, Ralstonia* and *Herbaspirillum* (Supplementary Fig. S1). Despite the fact that such taxonomic groups have been reported in different breastmilk studies, including in a cohort of Mexican individuals^5,8,24,25^, we decided to remove them from our analysis.

The candidate division WPS-2 was present in 2.1% of the total relative abundance in our samples. This phylum has been described in human and canine oral microbiota and in soil^33–35^. WPS-2 was incorporated in the Human Oral Microbiome database (HOMD) in 2014^36^. While it has not been described in the microbiota of the newborns’ oral cavity, a study shows that WPS-2 was present in nasopharygeal samples from infants under 6 months of age^37^. This is the first time that WPS-2 is reported with high prevalence in breastmilk samples, which could be explained by the retrograde flux theory since we missed to register information regarding the number of times the newborn had been fed prior to sampling^6,38^.

In addition, we determined that colostrum microbial diversity was specific to the newborn gender (p ≤ 0.05). Our results are in accordance with previous reports in which maternal BMI and neonate gender were related to enrichment of *Staphylococcus*, *Stenotrophomonas* and *Burkholderia*^5^. However, *Staphylococcus* and *Stenotrophomonas* showed differential abundance according to the type of delivery, which might indicate a source of background contamination or carryover. Other reports suggest that the absence of physiological stress or hormonal signals could influence the microbial transmission process to milk^17,39^. Regardless of the pathology (GDM or obesity), female-related colostrum samples showed higher alpha diversity compared to the male subgroups, suggesting a more diverse microenvironment (Fig. 2A–C). It has been demonstrated that gut and oral microbiota from children and maternal breastmilk biochemical composition differ between female and male infants, possibly due to variation in hormone recruitment and energetic demand during pregnancy^5, 40–43^. We therefore strongly suggest that the neonate microbiota sex-bias should be an important consideration for the design of further experiments attempting to explain casualty in microbial changes due to any pathology.

On the other hand, microbial diversity in colostrum samples from women with obesity is still controversial, with both positive and negative associations^7,17–19^. This variability can be attributed to differences in study populations (geographical location, diet, socio-economic status), sample collection at different stages of lactation, neonate sex bias and microbial undetected unknown diversity^44^. For instance, it has been observed that higher diversity and numbers of *Lactobacillus* and *Staphylococcus* were related to breastmilk and intestinal microbiota in women with high BMI^17–19,45^. In addition, decreased *Streptococcus* abundance has been reported in breastmilk from subjects with high BMI (> 25 kg/m^2^) in Mexican–American mothers^7^. In our study, we observed that *Streptococcus* has lower relative abundance but higher diversity (number of ASVs) in the Ob-M (167ASVs) and the Ob-F (295 ASVs) subgroups. However, other reports show that colostrum samples from mothers with obesity presented a less diverse microbiota compared to samples from non-obese mothers^17^. Despite the sample heterogeneity, we guided our analysis with a general linear model to show that colostrum samples of individuals with obesity (Ob-F and Ob-M) or GDM (GD-F and GD-M) are enriched for *Staphylococcus* and *Prevotella*, respectively (p ≤ 0.10) (Fig. 3A and Supplementary Table S2)*.*

Complementarily, we implemented the Aldex2 tool, which performs a log transformation and replacement of the zero values in the obtained results to create a matrix that allows determination of significant differences of taxa between subgroups^27^. Our results showed a differential presence of *Bdellovibrionaceae* and *Saccharimonadales*, which are ultra-small parasitic bacteria, in the subgroup Ob-M compared to NW-M (Fig. 4B). While further research is required, this pattern can be attributed to resilience mechanisms of the breastmilk microenvironment to maintain a functional equilibrium through specific predatory interactions with gram-negative bacteria such as *Burkholderia* and *Chryseobacterium*, which also appear to be present in higher proportions in the Ob-M subgroup (Fig. 3A and Supplementary Table S2). This may be explained by the detection of DNA fragments resulting from bacterial lysis. *Bdellovibrionaceae* has been found in soil, freshwater and the human gut from both healthy subjects and those suffering from inflammatory diseases^46,47^. This taxon is considered as a potential probiotic, since it could modulate gut biodiversity through predation of bacteria correlated in chronic inflammatory diseases, such as obesity and Crohn’s disease^48,49^. On the other hand, *Saccharimonadales* has been reported in the human oral cavity, intestines, skin, and female genital tract^50,51^. Lif et al.^52^ related the impact of birthing method and a higher prevalence of this novel phylum in oral biofilm samples of infants delivered vaginally compared to those born by cesarean section. Although *Saccharimonadales* remains difficult to culture, its presence in adult subgingival plaque, vagina and colon has been associated with human inflammatory mucosal diseases^50,53,54^.

We observed differential prevalence of *Burkholderiaceae* and *Sphingobacteriaceae* in colostrum samples from the Ob-M subgroup compared to its female counterpart (Ob-F; Fig. 4C). Similar results reported in oral samples from male infants, since they reported a higher abundance of *Brachymonas* and *Sphingomonas*^42^. We hypothesize that differences in colostrum microbiota according infant gender may influence the conditioning of the neonate gut microenvironment for bacterial communities related to the metabolism of nutrients involved in sex-related neurodevelopment^41^.

We observed a higher relative abundance of *Staphylococcus* and *Prevotella* in both GDM subgroups compared to their corresponding controls. Similar profiles have been reported for gut microbiota in individuals with GDM^22,55,56^. On the other hand, a high prevalence of the proinflammatory *Prevotella* has been observed in the oral cavity, amniotic fluid, vagina and gut microbiota of pregnant women with GDM, which reinforces vertical mother-to-baby transmission and supports the enteromammary theory of breastmilk microbiota origin^21,55,57–59^. *Rhodobacteraceae* was observed in higher proportion in the GD-F compared to the NW-F subgroup (adjusted p-value < 0.10). *Rhodobacteraceae* has mostly been reported in soil^60^, but also in the breastmilk of healthy Mexican women^24^, human skin^61^, meconium^62^ and fecal samples from patients suffering from diarrhea^63^. Functional shotgun metagenomic approaches are required to determine the strain-level diversity and the role of specific taxa including fungi and virus in order to develop hypothesis of keystone members of the community under the specific microenvironment shaped by GDM and obesity.

**Figure 5:**
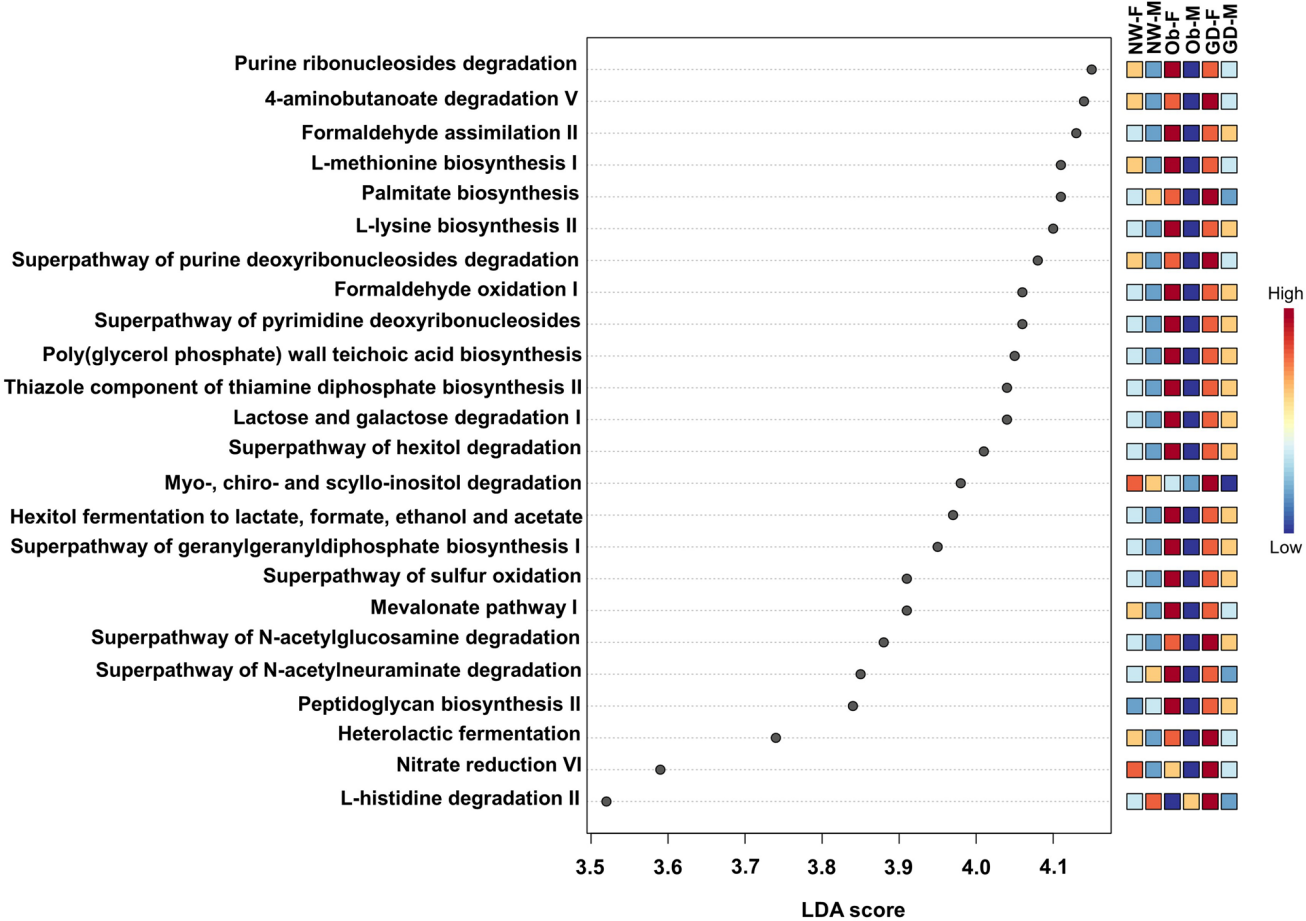
LEfSe analysis of the colostrum’s functional profiling prediction between subgroups. A significance p-value ≤ 0.05 and an effect size threshold of 3.5 were used for all Metacyc pathways evaluated. Graphic was generated in MicrobiomeAnalyst web-based platform^80^. *NW-F* healthy normal weight-female, *NW-M* healthy normal weight-male, *Ob-F* obesity positive GDM negative-female, *Ob-M* obesity positive GDM negative-male, *GD-F* GDM positive obesity negative-female baby, *GD-M* GDM positive obesity negative-male.

To attempt to provide a preliminary functional insight for the taxonomic profiles observed in our study, we used the PICRUSt2 software^64^ and Linear Discriminant Analysis (LDA) Effect Size (LEfSe)^65^. A total of 24 metabolic pathways reported in the MetaCyc database^66^ were considered significant (p-value < 0.05, LDA score > 3.5). While the results suggest that bacterial pathways involved in carbohydrate metabolism were significantly represented in the obese subgroups, the amino acid biosynthesis pathways were overrepresented in the GDM subgroups (Fig. 5). Higher levels of branched-chain amino acids (BCAA) valine, leucine and isoleucine have been detected in blood from women with GDM and are linked to an increased risk of glucose intolerance, early pathogenesis of type 2 diabetes and a high relative abundance of intestinal pro-inflammatory species of *Prevotella*^67–70^. The role of *Prevotella* spp. in human health is controversial and depends on strain-level diversity since their presence and complex carbohydrate metabolism is affected by diet and lifestyle^67^. Lactation for more than 3 months in women with GDM has been shown to decrease the risk of developing type 2 diabetes, which is associated with a reduction in the BCAA biosynthetic pathways in breastmilk. Further research is required in order to explain the molecular mechanism and the role of the microbiota of this phenotype^71^. While preliminary, our functional analysis suggests that palmitate biosynthesis and inositol isomer degradation pathways were more prevalent in the GD-F subgroup. Palmitic acid is one of the major saturated fatty acids in colostrum. It provides around 25% of the fatty acids in milk and is involved in absorption of fat and calcium^72^. Mothers with GDM and obesity have been associated with higher levels of this free fatty acid in the placenta, umbilical cord and breastmilk^73–75^. On the other hand, myoinositol plays an important role in several biological processes related to cell survival, lipid metabolism, glycemic control and restoration of ovulation^76^. Women with GDM or polycystic ovary syndrome are deficient in inositol isomer biosynthesis^77,78^. Supplementation with myoinositol during pregnancy improves glucose metabolism, reduces the incidence and severity of GDM and decreases adverse neonatal outcomes^76–79^. We acknowledge that predictions of bacterial functional metabolic pathways based on 16S amplicon sequencing are biased towards existing reference genomes and caution must therefore be used in the interpretation of these results^64^.

The factors determining colonization of the infant's gut microbiota are important and could lead to improved health policies. Amplicon sequencing using NGS technologies is among the most reliable methods for large-scale microbial compositional studies^81^; however, for breastmilk studies, clinical protocols including detailed demographic and lifestyle information such as detailed dietary habits, number of feeds prior to sampling, and sex of the infant, antibiotic usage could facilitate identification of differential taxa amongst the study groups. Our study indicates that neonate gender bias is an important factor to be considered in all breastmilk microbiota studies, since it influences the prevalence of certain bacteria such as *Streptococcus, Xanthobacteraceae* and *Burkholderia*. We also identified that GDM is related to a higher microbial diversity with overrepresentation of *Prevotella* and significantly overrepresented by amino acid and carbohydrate metabolism bacterial pathways. While *Prevotella* is a relevant taxonomic group within the microbial population of colostrum samples from individuals with GDM, *Staphylococcus*, *Corynebacterium* 1 and *Anaerococcus* are overrepresented in the colostrum of participants with obesity. However, this finding was specific to the sex of the newborn and affected by the implementation of peripartum antibiotics, and further studies of the functional role of such taxa are key in order to understand the dynamics of the establishment of the infants' gut microbiota under the influence of obesity and GDM. Such studies are required for the potential design of probiotics and in the search of possible therapeutic agents contributing to the homeostasis of the infant's gastrointestinal immune system.

## Material and methods

### Ethical statements and study population

The study protocol was approved by institutional Review Boards at the Escuela de Medicina y Ciencias de la Salud, Tecnológico de Monterrey, with the ID P000185-CarMicrobioLHum2018-CEIC-CR002, on May 6th, 2019. The study was conducted according to the ethical standards of the institutional committee, and in compliance with the Declaration of Helsinki. Recruitment was conducted at Hospital Regional Materno Infantil between May and December of 2019. Sample size was initially set to include 50 participants based on initial project budget. We included mother-infant pairs, within 20 and 32 years of maternal age, and with verified addresses in the Monterrey Metropolitan Area. All mothers accepted the invitation to participate and signed the informed consent document. Exclusion criteria were: mothers who had a history of antibiotic usage in the 3 months prior to delivery; mothers who had prolonged exposure to antibiotics (more than 3 weeks) at any time during pregnancy; mothers who received immunosuppressive or immunomodulatory corticosteroid therapy; history of vegan, ovolactovegetarian or exclusion diet (e.g.: ketogenic diet); history of bariatric surgery or any complicated surgery; history of feeding disorders; exposure to antineoplastic drugs, histamine-H2 receptor antagonists or proton pump inhibitors and/or monoclonal antibodies; and/or history of diarrhea during the three weeks prior to delivery. In addition, those with an uncertain last menstrual period date or irregular periods that gave place to an uncertain pregnancy dating were excluded. Elimination criteria were: antibiotics for more than 24 h post-delivery; need for intensive care (mother or infant) and/or any condition that impeded collection of the breastmilk.

Those selected mother-infant pairs were allocated for further analysis to one of the three study groups, according to their BMI and health condition: 12 obese women (BMI ≥ 30 kg/m^2^), 13 women suffering from gestational diabetes (BMI ≤ 29.9 kg/m^2^) and 18 control healthy women (BMI ≤ 25 kg/m^2^). The study groups were further sub-divided according to the sex of the baby.

### Sample collection and processing

After gentle cleansing of the breasts with sterile water only, each mother performed a gentle circular massage of each breast until a few drops of colostrum appeared. These first drops were disregarded, and the mother self-expressed approximately 5 mL (when possible) of colostrum into a sterile falcon-type 20 mL polypropylene tube, under close medical supervision. The procedure was repeated on the other breast. Extreme care was taken to avoid contact between the milk and the breast skin or fingers. The tubes were then closed, and kept at -20 °C for no more than 48 h until DNA extraction.

### DNA extraction

Genomic DNA was extracted from 1 mL of colostrum using an optimized phenol–chloroform protocol^82^. Samples were thawed on ice and centrifuged at 16,000×*g* for 15 min. The fat rim was then carefully removed, and PBS washes were performed to eliminate fat residues (0.5 mL sterile PBS), centrifugation at 16,000×*g* for 10 min). The pellet was resuspended in 0.5 mL of extraction buffer (2 mM Tris–HCl pH 8, 0.2 mM EDTA, 20 mM NaCl, 0.4% Triton X-100, 0.2% SDS) and 0.3 mL of 3 M of sodium acetate. An additional step of mechanical lysis was performed by bead-beating with lysing matrix A using a FastPrep (MP Biomedicals, Santa Ana, CA) disruptor at a speed setting of 5.5 m/s for 25 s. The lysate was submitted to enzymatic lysis with 10 µL of proteinase K (10 mg/mL), 5 µL of lysozyme (10 mg/mL) and 10 µL of RNAse, and incubated at 60 °C for 1 h. After incubation, 100 µL of 1.5 M NaCl (filter sterile) were added and carefully mixed and maintained at room temperature for 5 min. Following incubation, the mixture was centrifuged at 16,000×*g* for 15 min and the supernatant transferred into a new tube and extracted twice with an equal volume of phenol : chloroform:isoamyl-alcohol (25:24:1). The DNA was precipitated through the addition of 0.6 volumes of isopropanol and incubation at − 80 °C for 1 h. The samples were then centrifuged (16,000×g for 15 min), and the isopropanol removed. The pellet was washed twice with 70% ethanol, air dried and resuspended in pre-heated 50 µL of nuclease-free water. The DNA was measured using a NanoDrop ND-1000 UV spectrophotometer (Thermo Fisher Scientific, Waltham, MA, USA) and DNA integrity was confirmed through agarose gel electrophoresis. Unless otherwise specified, all reagents were purchased from Sigma Aldrich.

In order to identify any contaminants during the DNA extractions, we processed all of the samples in a total of 16 different randomized batches using freshly prepared solutions prior to running the experiments^23^. Microbial compositional information for all batches is provided as Supplementary Fig. S1.

### DNA sequencing and analysis

DNA samples were sequenced at the Advanced Genomics Unit (LANGEBIO, CINVESTAV) using Illumina MiSeq (2 × 300) following the 16S rRNA amplicon sequencing library preparation (as per manufacturer recommendations) for amplification of the V3–V4 hypervariable region with the universal primers 341F 5′CCTACGGGNGGCWGCAG3′ and 785(R) 5′GGACTACHVGGGTATCTAATCC 3′. We normalized the DNA for sequencing at 25 ng/μl.

Bioinformatic analyses were carried out using QIIME 2 v.2019.7^83^. Sequencing readings were quality filtered using the q2-demux plugin with a minimum length of 270 nucleotides followed by denoising with DADA2^84^. Single-paired filtered readings were used for the taxonomic species profile using amplicon sequence variants (ASVs) with the q2-feature-classifier^85^ against the Silva 132 database with a sequence identity limit set at 99%^86^. Removal of potential contaminants included ASVs belonging to *Cyanobacteria*, *Phyllobacterium*, *Chloroflexi*, mitochondria/chloroplast and rare taxa (with less than 25 reads across the entire dataset). The resulting ASVs were aligned with mafft v2019.7^87^ and implemented to create a phylogeny with fasttree2 v2019.7^88^. Rarefaction of sequences to 6130 per sample was used to perform alpha and beta diversity analyses. Observed ASVs, Shannon index and Faith’s phylogenetic diversity were used as alpha-diversity metrics; UniFrac (weighted and unweighted) and robust Aitchison distances were used for the creation of PCoA and RPCA, respectively. We determined bacterial families present in all samples with a minimum relative abundance of 1% overall as a core microbiota. ASVs that were assigned at family level were considered to perform differential abundance analysis using the Aldex2 tool v1.14.1^27^. We implemented the Phylogenetic Investigation of Communities by Reconstruction of Unobserved States 2 (PICRUSt2) v2.3.0-b software^64^ with default options (picrust pipeline.py) to determine the potential link between the microbiome environment and the functional metabolism based on metabolic pathways reported in the MetaCyc database^66^. The raw data was deposited and is available at the NCBI Sequence Read Archive (SRA) under SRA accession number PRJNA638389.

### Data processing

QIIME2 α-diversity outputs and ASVs count tables at the family and genus taxonomic level were imported and processed in Minitab 19. Association of observed ASVs, Shannon index, Faith’s phylogenetic distance and transformed ASVs count tables with maternal health condition, mode of delivery, antibiotic exposure, parity and gender of the neonate was assessed by general linear model (GLM) with a p-value of ≤ 0.10. β-Diversity significance for UniFrac and robust Aitchison distances was calculated using permutational ANOVA (PERMANOVA) with 999 permutations. Differential bacteria were assessed with Welch’s t test with a Benjamini-Hochberg's false discovery rate (FDR) p-value correction after a centered log ratio (CLR) transformation, with zero-replacement of taxa counts. Functional metabolism prediction results by PICRUST2 were further analyzed in the Microbiome Analyst web-based platform^80^ using the LEfSe method^65^ which performs a Kruskal–Wallis rank sum test to determine the significantly different features between groups and a linear discriminant analysis (LDA) to estimate their effect size. A metabolic pathway was considered significant with a p-value of ≤ 0.05 and an LDA score ≥ 3.5. The effect of the most different taxa (*Staphylococcus*, *Chryseobacterium*, *Rhizobiaceae*, *Streptococcus*, *Xanthobacteraceae*, *Rhizobium* and *Pseudacidovorax*) by newborn gender over the clustering of samples per subgroup was evaluated using Principal Component Analysis (PCA). Statistical analyses were performed in Minitab 19 and plotted in GraphPad Prism 6.

## Supplementary Information

Supplementary Information.

## Data Availability

Raw data is available in the NCBI under ID number 638389 and Bioproject accession number PRJNA638389.

## Abbreviations

GDM: Gestational diabetes mellitus
BMM: Breastmilk microbiota
ASV: Amplicon sequence variant
BMI: Body mass index
FDR: False discovery rate
NW-F: Healthy normal weight-female neonate
NW-M: Healthy normal weight-male neonate
Ob-F: Obesity positive GDM negative-female neonate
Ob-M: Obesity positive GDM negative-male neonate
GD-F: Gestational diabetes mellitus positive obesity negative-female neonate
GD-M: Gestational diabetes mellitus positive obesity negative-male neonate
BCAA: Branched-chain amino acids
GLM: General linear model
PCoA: Principal coordinate analysis
RPCA: Robust principal component analysis
PERMANOVA: Permutational ANOVA
PBS: Phosphate buffered saline
EDTA: Ethylenediaminetetraacetic acid
SDS: Sodium dodecyl sulfate
